# Propofol Improved Glucose Tolerance Associated with Increased FGF-21 and GLP-1 Production in Male Sprague-Dawley Rats

**DOI:** 10.3390/molecules25143229

**Published:** 2020-07-15

**Authors:** Chih-Cheng Wu, Chih-Jen Hung, Ya-Yu Wang, Shih-Yi Lin, Wen-Ying Chen, Yu-Hsiang Kuan, Su-Lan Liao, Ching-Ping Yang, Chun-Jung Chen

**Affiliations:** 1 Department of Anesthesiology, Taichung Veterans General Hospital, Taichung City 407, Taiwan; chihcheng.wu@gmail.com (C.-C.W.); hung@vghtc.gov.tw (C.-J.H.); 2 Department of Financial Engineering, Providence University, Taichung City 433, Taiwan; 3 Department of Data Science and Big Data Analytics, Providence University, Taichung City 433, Taiwan; 4 Department of Family Medicine, Taichung Veterans General Hospital, Taichung City 407, Taiwan; yywang@vghtc.gov.tw; 5 Institute of Clinical Medicine, National Yang Ming University, Taipei City 112, Taiwan; sylin@vghtc.gov.tw; 6 Center for Geriatrics and Gerontology, Taichung Veterans General Hospital, Taichung City 407, Taiwan; 7 Department of Veterinary Medicine, National Chung-Hsing University, Taichung City 402, Taiwan; wychen@dragon.nchu.edu.tw; 8 Department of Pharmacology, Chung Shan Medical University, Taichung City 402, Taiwan; kuanyh001@gmail.com; 9 Department of Medical Research, Taichung Veterans General Hospital, Taichung City 407, Taiwan; slliao@vghtc.gov.tw (S.-L.L.); d49407012@ym.edu.tw (C.-P.Y.); 10 Department of Medical Laboratory Science and Biotechnology, China Medical University, Taichung City 404, Taiwan; 11 Ph.D. Program in Translational Medicine, College of Life Sciences, National Chung Hsing University, Taichung City 402, Taiwan

**Keywords:** anesthetics, hyperglycemia, insulin resistance, propofol

## Abstract

Anesthetics, particularly volatile anesthetics, have been shown to impair glucose metabolism and cause hyperglycemia, closely linking them with mortality and morbidity as related to surgery. Beyond being an anesthetic used for general anesthesia and sedation, intravenous hypnotic propofol displays an effect on glucose metabolism. To extend the scope of propofol studies, its effects on glucose metabolism were evaluated in male Sprague-Dawley rats of various ages. Unlike chloral hydrate and isoflurane, propofol had little effect on basal glucose levels in rats at 2 months of age, although it did reduce chloral hydrate- and isoflurane-induced hyperglycemia. Propofol reduced postload glucose levels after either intraperitoneal or oral administration of glucose in both 7- and 12-month-old rats, but not those at 2 months of age. These improved effects regarding propofol on glucose metabolism were accompanied by an increase in insulin, fibroblast growth factor-21 (FGF-21), and glucagon-like peptide-1 (GLP-1) secretion. Additionally, an increase in hepatic FGF-21 expression, GLP-1 signaling, and FGF-21 signaling, along with a decrease in endoplasmic reticulum (ER) stress, were noted in propofol-treated rats at 7 months of age. Current findings imply that propofol may turn into insulin-sensitizing molecules during situations of existing insulin resistance, which involve FGF-21, GLP-1, and ER stress.

## 1. Introduction

Anesthetics are routinely administered to patients undergoing surgery and to critically ill patients for sedation. Clinically, the development of hyperglycemia in critically ill patients and patients undergoing surgery is associated with an increase in mortality, morbidity, and certain complications. For patients in the intensive care unit (ICU), the prevalence of hyperglycemia (>180 mg/dl) is 32.2% because of decreased responsiveness to the metabolic actions of insulin, named insulin resistance [1,2]. Volatile anesthetics, in particular halothane, isoflurane, and sevoflurane, cause impaired glucose tolerance, insulin resistance, and hyperglycemia, suggesting active roles in surgery- and critically ill-accompanied hyperglycemia [3,4,5].

**Figure 1:**
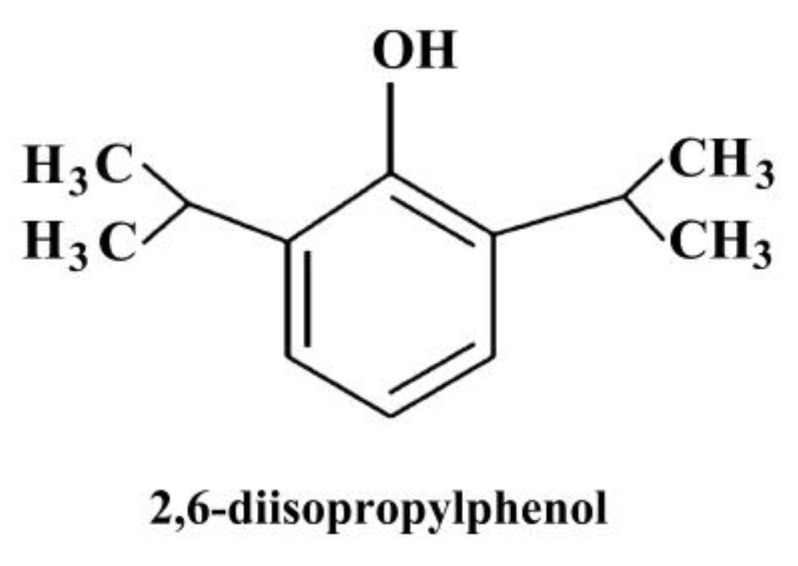
Chemical structure of propofol.

Propofol (2,6-diisopropylphenol) (Figure 1), an intravenous hypnotic agent, has been widely used for general anesthesia and sedation. Unlike volatile anesthetics, during clinical anesthesia with propofol, a relatively stable glucose homeostasis was noted [6,7]. However, experimental findings regarding propofol’s effects on glucose metabolism are contradictory. Anesthesia involving propofol in unstressed rats slightly induces insulin resistance due to the tumor necrosis factor α (TNF-α) elevation and acute lipid load [8,9,10,11,12], whereas in stressed rats and rhesus monkeys, propofol increases insulin secretion and improves glucose metabolism, and reduces TNF-α-induced hepatic insulin resistance [13,14,15]. Therefore, propofol has a regulatory effect on glucose metabolism, and its consequences are dependent upon a patient’s stress levels, environment, and disease status.

Glucagon-like peptide 1 (GLP-1), synthesized from enteroendocrine L cells after stimulation by nutrients, decreases postprandial blood glucose by promoting insulin secretion and reducing glucagon secretion [16]. Additionally, GLP-1 can stimulate the hepatic expression of fibroblast growth factor-21 (FGF-21), which plays a critical role in metabolic regulation, with beneficial effects on insulin resistance, hyperglycemia, hyperlipidemia, and obesity [17,18,19]. A study further indicated that FGF-21 can mediate effects of GLP-1 in attenuating hepatic glucose output [20], suggesting a link between GLP-1 and FGF-21 at the liver to regulate glucose homeostasis. However, whether or not FGF-21 and GLP-1 mediated the effects of propofol on glucose metabolism was less investigated.

Hyperglycemia is a risk factor for the prognosis of critically ill patients [1,2]. Since propofol is commonly used for sedation in patients in ICU, further elucidation of molecular mechanisms underlying the effects of propofol on glucose metabolism is important. The study first aimed to examine propofol’s effects on glucose dynamics and FGF-21 and GLP-1 expression using an animal model. It has been reported that age is a risk variable concerning glucose metabolism, and that the secretion of fasting GLP-1 and glucose-stimulated GLP-1 declines with aging [21,22,23]. Therefore, we also evaluated effects of age on FGF-21 and GLP-1 responses, to propofol, if any, in animals at various ages.

## 2. Results

### 2.1. Propofol Had Minimal Effect on Basal Glucose Level

**Figure 2:**
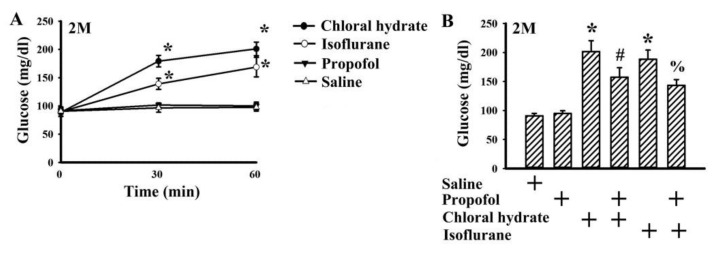
Propofol had minimal effect on fasting blood glucose. (**A**) The overnight fasting rats (2 months old) were administered inhaled isoflurane (4%) lasting for 1 h, or intraperitoneally injected with a bolus of chloral hydrate (400 mg/kg), propofol (50 mg/kg), or saline vehicle. Blood was collected from the tail veins at various times after treatments, and the levels of glucose were measured. (**B**) The overnight fasting rats (2 months old) were intraperitoneally injected with a bolus of propofol (50 mg/kg) or saline 10 min prior to a chloral hydrate (400 mg/kg) intraperitoneal injection or isoflurane (4%) inhalation. Additional blood was collected from the tail veins 60 min after treatments, and the levels of glucose were measured. All statistical data are expressed as mean values ± standard deviation. * *p* < 0.05 vs. saline group, # *p* < 0.05 vs. chloral hydrate group, and % *p* < 0.05 vs. isoflurane group, *n* = 6.

To determine the effects of anesthetics on fasting blood glucose, plasma levels of glucose were measured in anesthetized, conscious and unrestrained rats at 2 months of age. Rats anesthetized with intraperitoneally injected chloral hydrate (by 106.2%, *p* < 0.05) and inhaled isoflurane (by 73.0%, *p* < 0.05) experienced rising plasma levels of glucose, while the levels of those awake and intraperitoneally injected with propofol remained relatively constant (Figure 2A). Intriguingly, with an intraperitoneal bolus injection at a dose of 50 mg/kg, propofol alleviated the elevation of fasting blood glucose in rats anesthetized with chloral hydrate (by 21.8%, *p* < 0.05) and isoflurane (by 23.9%, *p* < 0.05) in rats at 2 months of age (Figure 2B).

### 2.2. Propofol Improved Impaired Glucose Tolerance

**Figure 3:**
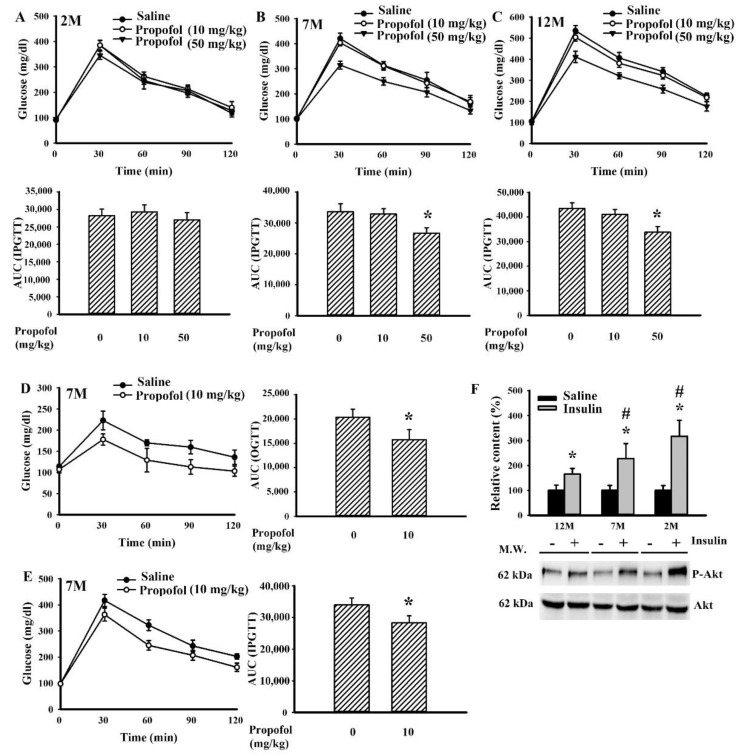
Propofol improved impaired glucose tolerance. The overnight fasting rats ((**A**) 2 months of age; (**B**) 7 months of age; (**C**) 12 months of age) were intraperitoneally injected with a bolus of propofol (10 and 50 mg/kg) or saline 10 min prior to an intraperitoneal injection of glucose solution (2 g/kg). Blood was collected from the tail veins at various times after treatments and the levels of glucose were measured. The total area under curve (AUC) of the glucose-time curves was calculated. (**D**) The overnight fasting rats (7 months of age) were intravenously injected with a bolus of propofol (10 mg/kg) or saline 10 min prior to an oral administration of glucose solution (2 g/kg). Blood was collected from the tail veins at various times after treatments, and the levels of glucose were measured. The AUC of the glucose-time curves was calculated. (**E**) The overnight fasting rats (7 months of age) were intravenously injected with a bolus of propofol (10 mg/kg) or saline 10 min prior to an intraperitoneal injection of glucose solution (2 g/kg). Blood was collected from the tail veins at various times after treatments, and the levels of glucose were measured. The AUC of the glucose-time curves was calculated. (**F**) The overnight fasting rats (2 months of age; 7 months of age; 12 months of age) were intraperitoneally injected with a bolus of insulin (10 U/kg) or saline 15 min prior to tissue preparation. The dissected liver tissues were subjected to protein extraction and the Western blot with indicated antibodies. One representative blot and quantitative data are shown. All statistical data are expressed as mean values ± standard deviation. * *p* < 0.05 vs. saline group and # *p* < 0.05 vs. Insulin group (12 M), *n* = 6.

The effects of propofol on postprandial glucose dynamics were then evaluated in rats of different ages. Under anesthesia with propofol, rats displayed lower postprandial blood glucose after the intraperitoneal glucose injection, in those at 7 months of age and 12 months of age but not 2 months of age (Figure 3A–C). Propofol is an intravenous hypnotic agent; administering an intravenous injection of propofol at a bolus dose of 10 mg/kg reduced postload glucose levels after the oral (Figure 3D) and intraperitoneal (Figure 3E) glucose administration in rats at 7 months of age. To find the insulin responsiveness amongst rats at 2, 7, and 12 months of age, the levels of Akt phosphorylation after insulin challenge were assessed in the liver tissues. Rats at 2 months of age (by 217% increase, *p* < 0.05) exhibited an apparent insulin response, while those at 12 months of age (by 65% increase, *p* < 0.05) displayed the least response (Figure 3F). The findings indicate that rats at 7 and 12 months of age showed decreased insulin responsiveness in reducing blood glucose levels and developed insulin resistance when compared with rats at 2 months of age.

### 2.3. Propofol Increased the Circulating Level of Fibroblast Growth Factor-21 (FGF-21), Glucagon-like Peptide-1 (GLP-1), and Insulin

**Figure 4:**
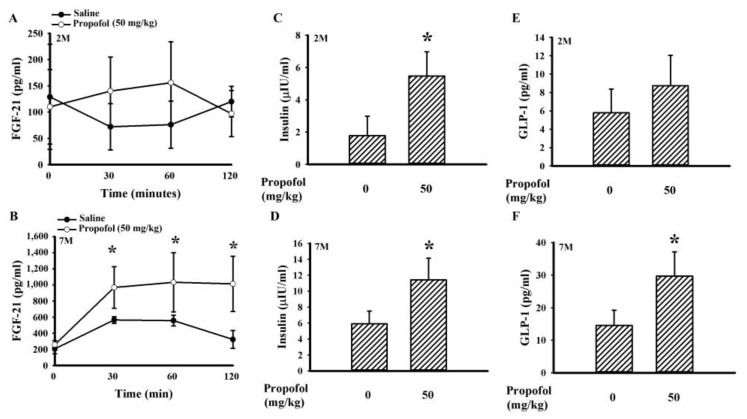
Propofol increased fibroblast growth factor-21 (FGF-21), insulin, and glucagon-like peptide-1 (GLP-1) secretion. The overnight fasting rats ((**A**,**C**,**E**) 2 months old; (**B**,**D**,**F**) 7 months old) were intraperitoneally injected with a bolus of propofol (50 mg/kg) or saline 10 min prior to an intraperitoneal injection of glucose solution (2 g/kg). Blood was collected from the tail veins at various times after treatments, and the levels of FGF-21 were measured (**A**,**B**). Blood was then withdrawn from the femoral artery 30 min after treatments, and the levels of insulin (**C**,**D**) and GLP-1 (**E**,**F**) were measured. All statistical data are expressed as mean values ± standard deviation. * *p* < 0.05 vs. saline group, *n* = 6.

Insulin plays a crucial role in glucose metabolism, and its secretion and action can be promoted by both GLP-1 and FGF-21 [17,18]. During the course of intraperitoneal glucose tolerance test (IPGTT), intraperitoneal injections of propofol had little effect on the circulating levels of FGF-21 in rats at 2 months of age (Figure 4A), while they did cause elevating plasma levels of FGF-21 (by 231.9% increase, *p* < 0.05) in rats at 7 months of age (Figure 4B) when compared with the saline vehicle groups. Thirty minutes after the intraperitoneal injection of glucose, the propofol treatment showed elevated insulin levels in rats at both 2 (by 207.8% increase, *p* < 0.05) (Figure 4C) and 7 (by 93.2% increase, *p* < 0.05) (Figure 4D) months of age. However, the elevation of plasma levels of GLP-1 was only noted in rats at 7 months of age (by 104.8% increase, *p* < 0.05) (Figure 4E,F).

### 2.4. Propofol Upregulated FGF-21 Signaling in the Liver

**Figure 5:**
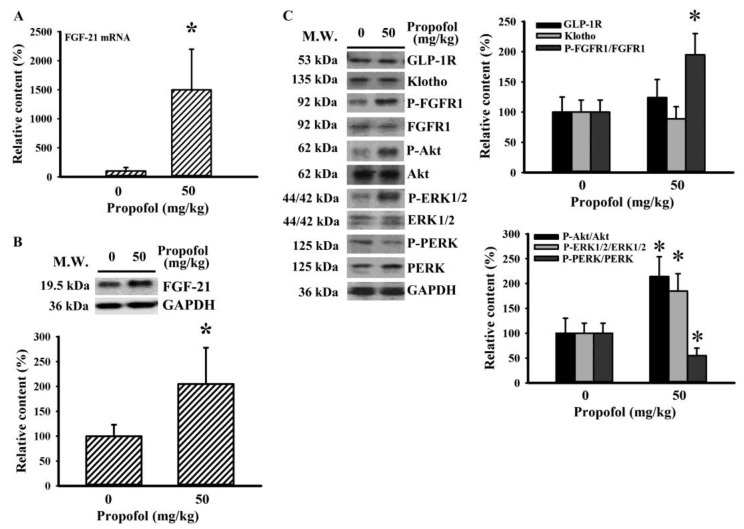
Propofol increased fibroblast growth factor-21 (FGF-21) and glucagon-like peptide-1 (GLP-1) signaling. The overnight fasting rats (7 months of age) were intraperitoneally injected with a bolus of propofol (50 mg/kg) or saline 10 min prior to an intraperitoneal injection of glucose solution (2 g/kg). (**A**) Total RNAs were extracted from the dissected liver tissues and subjected to real time reverse transcriptase polymerase chain reaction (RT-PCR) for the measurement of FGF-21 mRNA expression. (**B**,**C**) The dissected liver tissues were subjected to protein extraction and the Western blot with indicated antibodies. One representative blot and quantitative data are shown. All statistical data are expressed as mean values ± standard deviation. * *p* < 0.05 vs. saline group, *n* = 6.

FGF-21 is mainly synthesized in the liver [17]. Since the effect of propofol on FGF-21 was noted in rats at 7 months of age but not 2 months of age, the regulation of FGF-21 expression and action was further explored in rats at 7 months of age. At the time of 30 min after the intraperitoneal injection of glucose, propofol treatment increased hepatic FGF-21 mRNA (Figure 5A) and protein (Figure 5B) expression when compared with the saline control group. Accordingly, regulatory and signaling molecules surrounding FGF-21 expression and GLP-1 action were examined through Western blotting. Hepatic tissues expressed a relatively constant level of glucagon-like peptide-1 receptor (GLP-1R) and klotho independent of propofol treatment. However, propofol increased the phosphorylation level of fibroblast growth factor receptor-1 (FGFR1) (by 95% increase, *p* < 0.05), implying an action of FGF-21. Parallel elevations were observed in phosphorylated Akt (by 114% increase, *p* < 0.05) and extracellular signal-regulated kinase 1/2 (ERK1/2) (by 85% increase, *p* < 0.05). On the contrary, the level of protein kinase-like ER kinase (PERK) phosphorylation was alleviated by propofol (by 45% decrease, *p* < 0.05) (Figure 5C).

## 3. Discussion

Propofol is widely used for general anesthesia during surgery. Apart from its use as an anesthetic, accumulating evidence has suggested its neuroprotective potential [24,25,26,27]. A single bolus injection, continuous intravenous infusion, a single bolus injection followed by continuous intravenous infusion, or an intraperitoneal injection of propofol have been implicated in the prevention or treatment of neurological diseases [24,25,26,27]. The above-mentioned studies propose that apoptosis, autophagy, oxidative stress, inflammation, endoplasmic reticulum (ER) stress, mitochondria, neurotrophins, heme oxygenase-1 (HO-1), toll-like receptor-4 (TLR4), GABA, and PI3K/Akt may be action targets of propofol [24,25,26,27].

During an intra-peritoneal glucose tolerance test (IPGTT) or oral glucose tolerance test (OGTT), postprandial insulin secretion and insulin-sensitizing molecules play substantial roles in the uptake of postload glucose [16,17,18,19]. Propofol has been shown to increase the circulating level of insulin involving pancreatic ATP-sensitive K channels [9,13,14,28]. FGF-21 is an emerging insulin-sensitizing molecule, and its expression is under the control of food ingestion and an individual’s nutritional status. Upon engagement with FGFR1 receptor and the coreceptor β-klotho, FGF-21 can protect pancreatic β-cells while increasing insulin synthesis, induce adipose tissue secretion of adiponectin, and promote hepatic insulin sensitivity [17,18]. Herein, we found that the improvement of postload glucose levels caused by propofol, as seen in both the IPGTT (Figure 3B,C,E) and OGTT (Figure 3D), was accompanied by increased plasma levels of FGF-21 (Figure 4B) and insulin (Figure 4D). Additionally, the hepatic actions of FGF-21 were highly suggested by increased phosphorylation in FGFR1 and Akt (Figure 5C), downstream intracellular effectors of FGF-21 [17,18]. Furthermore, propofol alleviated hepatic ER stress, as evidenced by decreased PERK hyperphosphorylation (Figure 5C). Hepatic ER stress is closely linked with insulin resistance, and evidence indicates an alleviative effect of FGF-21 on hepatic ER stress [26,29,30]. Therefore, the secretion of functional FGF-21 is a putative surrogate of propofol in regulating glucose metabolism. Although the actions of the FGF-21 axis in propofol metabolic effects are suggested by current findings, its specific involvement requires further investigation using either knockout animals or FGF-21 sequester/inhibitor.

GLP-1 represents an alternative insulin-sensitizing molecule. GLP-1 production from enteroendocrine L cells is stimulated by food ingestion, and its stability is regulated by dipeptidyl peptidase IV (DPP-IV). The released GLP-1 induces glucose-stimulated insulin secretion, inhibits glucagon secretion, and protects pancreatic β-cells via GLP-1R [31]. Additionally, GLP-1 is also a stimulator of hepatic FGF-21 expression [17]. The elevation of FGF-21 (Figure 4B) and insulin (Figure 4D) secretion in propofol-treated rats was associated with higher GLP-1 secretion (Figure 4F) and increased hepatic ERK1/2 protein phosphorylation (Figure 5C); putative effector of GLP-1/GLP-1R [32,33]. The involvement of GLP-1 in the actions of propofol against glucose metabolism has been highlighted in the most current findings. It has been reported that cAMP and cAMP-response element binding protein (CREB) have substantial roles in GLP-1 synthesis and exocytosis, while protein phosphatase 2A (PP2A) has a negative effect on the action of cAMP/CREB [34,35]. Since propofol alleviates PP2A expression and activity [36], it inspires us to speculate that the axis of PP2A/cAMP/CREB could be a link between propofol and GLP-1/FGF-21. The proposed theory warrants further investigation.

Age is a critical factor contributing to both impaired glucose metabolism and insulin resistance [21,22]. The improved effects of propofol on glucose metabolism appeared to be closely linked with age when comparing findings from rats at 2, 7, and 12 months of age (Figure 3A–C). Unlike 7- and 12-month-old rats, hepatic tissues from 2-month-old rats showed a remarkable effectiveness to insulin responses (Figure 3F), while being relatively resistant to the actions of propofol (Figure 3A–C). Although they shared a similar elevation in plasma insulin (Figure 4C,D), the alterations in FGF-21 (Figure 4A,B) and GLP-1 (Figure 4E,F) were apparent only in rats at 7 and 12 months of age. However, propofol offered improved effects on chloral hydrate- and isoflurane-induced hyperglycemia in rats at 2 months of age (Figure 2B). Studies [14,15] reported on the improved effects propofol provides are against surgery- and TNF-α-induced hepatic insulin resistance. Although contradictory findings still remain in available literature showing an impairment of glucose metabolism by propofol in unstressed animals and hepatocytes [8,9,10,11,12], current findings imply that propofol may turn into insulin-sensitizing molecules upon situations of existing insulin resistance. However, this assumption requires a deeper investigation in order for a scientific judgment to be made.

In general anesthesia in humans, the suggested doses for maintenance of anesthesia are 3–12 mg/kg/h. The intravenous infusion of propofol at a dose of 50 mg/kg/h (44–55 mg/kg/h) in rats for maintenance of general anesthesia is equivalent to human receiving a dose of 8.1 mg/kg/h according to the guide for dose conversion between animals and human based on body surface area [12,27,37]. Administering a bolus dose of 10 mg/kg for intravenous injection, and 50 mg/kg for intraperitoneal injection, propofol reduced postload glucose levels after the intravenous and intraperitoneal glucose injections (Figure 3A–E). Unlike volatile anesthetics, propofol had little effect on basal glucose levels (Figure 2A), while improving glucose metabolism upon situations of insulin resistance (Figure 3, Figure 4 and Figure 5). These current findings highlight an alternative biological activity of propofol. However, before there is a translation into clinical practice, further investigations are required.

It should be noted that the molecular basis underlying propofol’s effects on glucose metabolism remains incomplete, in case of insulin resistance and insulin sensitivity. The consequences of repeated propofol exposure were not investigated. Besides, the inductive and action mechanisms of GLP-1 and FGF-21 by propofol still required further investigation. Although further study is still needed surrounding the roles of propofol in the changes of biochemical profiles under anesthesia, the results in our study can offer contribution in the choice and consideration of selection of anesthetics in the geriatric anesthesia, and this was seldom mentioned and reported in previous studies.

## 4. Materials and Methods

### 4.1. Animal Study

Male Sprague-Dawley rats at 2 months of age (*n* = 96 in total), 7 months of age (*n* = 48 in total), and 12 months of age (*n* = 24 in total) were purchased from BioLASCO Taiwan Co., Ltd. The rats were housed in a controlled animal facility for acclimatization (1 week). An IPGTT (*n* = 6 per time group) and an OGTT (*n* = 6 per time group) were performed in overnight fasting rats through the intraperitoneal and oral administration of glucose solution (2 g/kg body weight), respectively. Blood was then collected from tail veins at various postload times, and the levels of glucose were measured (hand-held Accucheck glucometer, Roche Diagnostics, Indianapolis, IN, USA). The total Area Under Curve (AUC) for both the IPGTT and OGTT was calculated. The conduction of animal studies strictly adhered to the Institute’s guidelines, and all procedures had been approved by the Animal Experimental Committee of Taichung Veterans General Hospital (IACUC No. La-1041316, Oct. 13, 2015). This study focused on the changes of blood glucose levels and other biochemical profiles under the presence of different anesthetics. The rats receiving a specific anesthetic were randomly selected from the same age of rats. Besides, the study designs and detailed animal allocations were blinded to the researchers, and they will not have expected results in the analysis. Furthermore, the results of the experiments were objectively presented by the analysis machines and recorded to minimize the bias in this study.

### 4.2. Blood Sample Reparation

Blood was withdrawn from the left femoral artery and plasma samples were kept at −80 °C until analyses (*n* = 6 per time group). The plasma levels of insulin (Shibayagi, Gunma, Japan), FGF-21, and GLP-1 (R&D Systems, Minneapolis, MN, USA) were measured using enzyme-linked immunosorbent assay (ELISA) kits, according to the manufacturer’s instructions.

### 4.3. Tissue Collection and Western Blot Analyses

The dissected liver tissues were stored in liquid nitrogen until analyses (*n* = 6 per group). Otherwise, insulin (0 and 10 U/kg) was intraperitoneally administrated 15 min prior to tissue collection in certain rats (*n* = 6 per group). The tissues were homogenized in tissue-protein extraction reagent (T-PER, Pierce Biotechnology, Rockford, IL, USA). After sodium dodecyl sulfate–polyacrylamide gel electrophoresis (SDS-PAGE) separation and transfer, the polyvinylidene fluoride (PVDF) membranes reacted with primary antibodies, horseradish peroxidase-labeled IgG, subsequently followed by Enhanced Chemiluminescence (ECL) detection reagents. The intensity of recognized protein was quantified through densitometry. The primary antibodies recognized included GLP-1R (1:1000, sc-390774), Klotho (1:500, sc-515939), Akt (1:2000, sc-8312), phospho-Akt (1:1000, sc-7985-R), ERK1/2 (1:2000, sc-514302), phospho-ERK1/2 (1:1000, sc-7383), PERK (1:1000, sc-9479), phospho-PERK (1:1000, sc-32577), FGF-21 (1:500, sc-16842), glyceraldehyde-3-phosphate dehydrogenase (GAPDH, 1:2000, sc-32233) (Santa Cruz Biotechnology, Santa Cruz, CA, USA), FGFR1 (1:1000, #9740), phospho-FGFR1 (1:1000, #3471) (Cell Signaling, Danvers, MA, USA).

### 4.4. RNA Preparation and Quantitative Real-Time Reverse Transcriptase Polymerase Chain Reaction (RT-PCR)

The liver tissues were stored in liquid nitrogen until analyses (*n* = 6 per group). A TriZol RNA isolation reagent (Invitrogen, Carlsbad, CA, USA) was used to extract total RNAs from the tissues, and the obtained RNAs were subjected to cDNA synthesis and PCR reaction (ABI StepOne^TM^, Applied Biosystems, Foster City, CA, USA). The levels of gene expression were calculated using the ΔΔCT method. The first-strand cDNA was synthesized by adding 1 μg RNA and 0.25 μg random primers. PCR primers (0.4 μM) were as follows: FGF-21, 5′-CACCGCAGTCCAGAAAGTCT and 5′-CATAGAGAGTTCCATCTGGTTGTTG; and β-actin, 5′-AAGTCCCTCACCCTCCCAAAAG and 5′-AAGCAATGCTGTCACCTTCCC.

### 4.5. Statistical Analysis

All statistical analyzes were performed using SPSS (version 12.0; SPSS, Chicago, IL, USA). The *t* test was applied for comparisons between two groups, while posthoc comparisons among groups after one-way analysis of variance (ANOVA) were performed using the Tukey test or the Dunnett test. The statistical significance levels were set to *p* < 0.05. All statistical data are expressed as mean values ± standard deviation.
